# Hydrophobins—Unique Fungal Proteins

**DOI:** 10.1371/journal.ppat.1002700

**Published:** 2012-05-31

**Authors:** Jagadeesh Bayry, Vishukumar Aimanianda, J. Iñaki Guijarro, Margaret Sunde, Jean-Paul Latgé

**Affiliations:** 1 Institut National de la Santé et de la Recherche Médicale Unité 872, Paris, France; 2 Université Pierre et Marie Curie – Paris 6, UMR S 872, Centre de Recherche des Cordeliers, Equipe 16-Immunopathology & Therapeutic Immunointervention, Paris, France; 3 Université Paris Descartes, UMR S 872, Paris, France; 4 Institut Pasteur, Unité des Aspergillus, Paris, France; 5 Institut Pasteur, Unité de RMN des Biomolécules, Paris, France; 6 CNRS UMR 3528, Paris, France; 7 University of Sydney, Discipline of Pharmacology, New South Wales, Australia; Duke University Medical Center, United States of America

Microorganisms are often covered by a proteinaceous surface layer that serves as a sieve for external molecular influx, as a shield to protect microbes from external aggression, or as an aid to help microbial dispersion. In bacteria, the latter is called the S-layer, in Actinomycetes, the rod-like fibrillar layer, and in fungi, the rodlet layer [1]. The self-assembly properties and remarkable structural and physicochemical characteristics of hydrophobin proteins underlie the multiple roles played by these unique proteins in fungal biology.

## What Are Hydrophobins?

**Figure 1:**
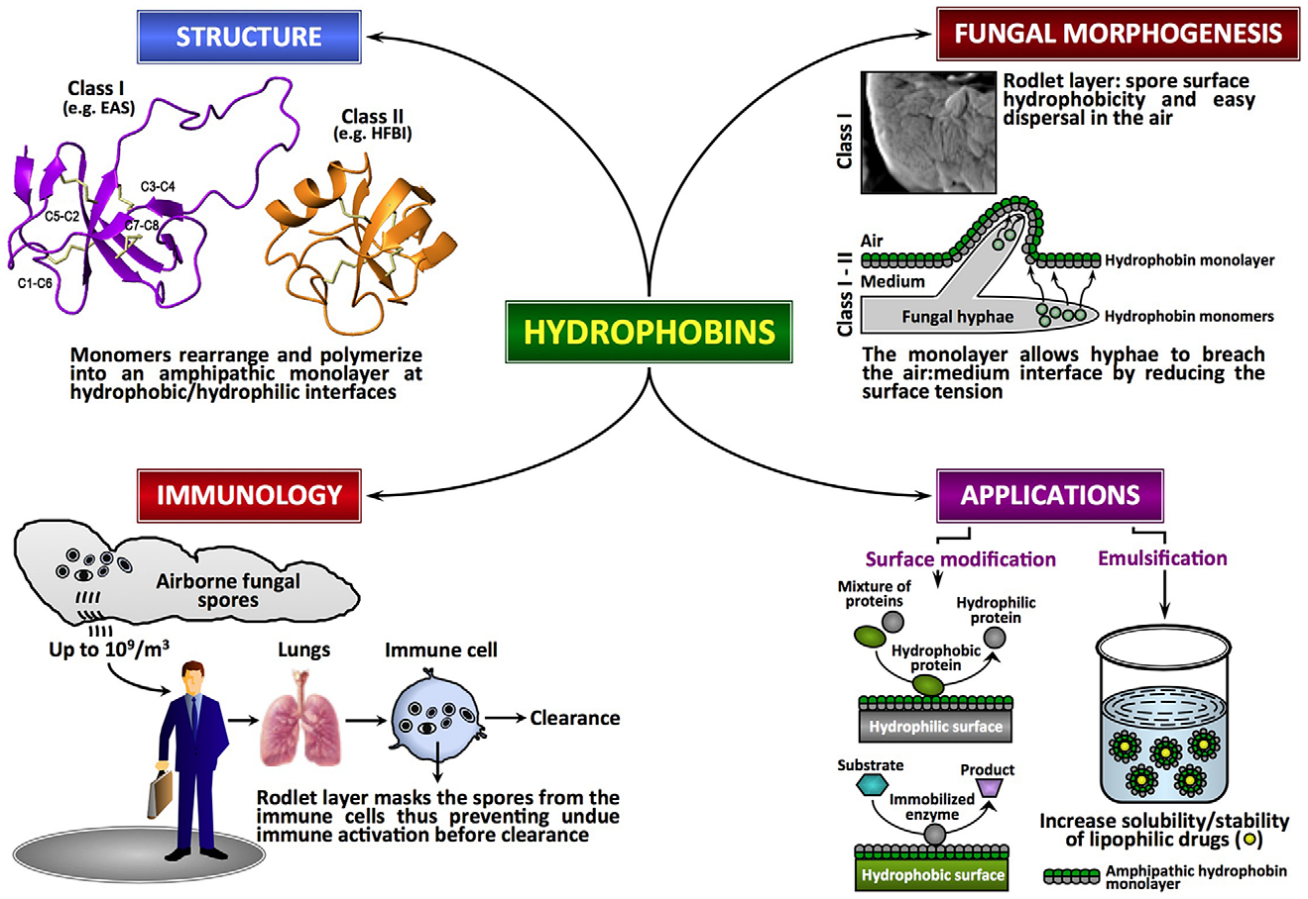
Fungal hydrophobins. Fungal hydrophobins are unique amphipathic proteins with multiple roles in the fungal life cycle and in mediating interactions between fungus and host. There is diversity in the primary sequences of hydrophobins but they share a similar core three-dimensional structure and a pattern of four disulfide bonds (shown in amber) that stabilize the structures. Increasingly, these proteins show potential for modification of hydrophobic nanomaterials and in solubilizing lipophilic drugs.

Hydrophobins, low molecular mass (≤20 kDa) secreted proteins of fungi, are characterized by moderate to high levels of hydrophobicity and the presence of eight conserved cysteine (Cys) residues. These proteins are able to assemble spontaneously into amphipathic monolayers at hydrophobic–hydrophilic interfaces. Although functional homologues are reported in *Streptomyces* (chaplins, SapB, and SapT for aerial morphogenesis; [2]), hydrophobins are unique to the fungal kingdom. Fungal genome analyses have indicated that hydrophobins generally exist as small gene families with two to ten members, although certain species contain more members (e.g., *Coprinus cinereus* displays 33 members; http://www.broadinstitute.org) [3], [4]. Hydrophobins show very little sequence conservation in general, apart from the idiosyncratic pattern of eight Cys residues implicated in the formation of four disulfide bridges (Cys1–Cys6, Cys2–Cys5, Cys3–Cys4, Cys7–Cys8) [5] (Figure 1). Based on hydropathy plots, solubility and the type of layer they form, hydrophobins are divided into two classes [6, Reference S1 in Text S1], although recent bioinformatics studies suggest that intermediate/different forms can also exist and that many hydrophobins with distinct physicochemical characteristics may have been overlooked in the past [4], [7]. In class I, considerable variation is seen in the inter-Cys-spacing; these hydrophobins assemble into highly insoluble polymeric monolayers composed of fibrillar structures known as rodlets. The rodlets are extremely stable, can only be solubilized with harsh acid treatments, and the soluble forms can polymerize back into rodlets under appropriate conditions. Despite the low sequence similarity, class I hydrophobins from different fungal species could partially complement a *Magnaporthe grisea* class I hydrophobin gene (*MPG1*) deletion mutant, suggesting that hydrophobins constitute a closely related group of morphogenetic proteins [8]. The sequence and the inter-Cys spacing are more conserved in class II; the monolayers formed by class II hydrophobins lack the fibrillar rodlet morphology and can be solubilized with organic solvents and detergents.

## Hydrophobins at the Interface in the Fungal Life Cycle

Fungi are heterotrophic terrestrial eukaryotes, showing two types of growth morphologies: unicellular yeast and multicellular filamentous forms. Yeasts are hydrophilic and they lack hydrophobins. The vegetative hyphae of filamentous fungi growing on moist environments are also hydrophilic and do not show the presence of rodlets on their surface. In contrast, the aerial hyphae and the asexual spores (conidia) are hydrophobic, due to the presence of hydrophobins. The functions of hydrophobins are related to their high surfactant activity, which results from their self-assembly at hydrophilic–hydrophobic interfaces to form an amphipathic monolayer. The hydrophobin layer reduces the surface tension of the medium or the substratum in/on which fungi grow, allowing them to breach the air–water interface or preventing water-logging while maintaining permeability to gaseous exchange [9]. Spores produced on the aerial structures of filamentous fungi are covered by a hydrophobin rodlet layer that renders the conidial surface hydrophobic and wet-resistant, thus facilitating spore-dispersal in the air. The rodlet-forming hydrophobins are essential for these fungi to complete their biological cycle. In many “wet” fungi (e.g., *Conidiobolus obscurus*), the rodlet-layer is covered by a mucilaginous extracellular matrix that helps the conidia to bind to the substrate, and once the spores are bound to the host, the rodlet-layer is unmasked for better resistance to the environment [10]. In the basidiomycete *Agaricus bisporus*, the hydrophobin HypA, found in the peel tissue of the mushroom cap, is suggested to form a protective layer during fruiting body development [11]. In *Cryphonectria parasitica*, the deletion of the gene coding the class II hydrophobin cryparin generated a mutant incapable of erupting through the bark of the tree [12]. Hydrophobins are also reported to play a role in the surface interaction during infection-related development of *M. grisea* [13, Reference S2 in Text S1]. In the symbiotic phenotypes of lichen-forming ascomycetes *Xanthoria* spp., the continuous rodlet-layer seals the apoplast continuum [14].

## Structure of Hydrophobins

Hydrophobins from both classes have been studied *in vitro* and have been shown to be highly surface active and to form amphipathic monolayers on hydrophobic/hydrophilic surfaces. The crystal structures of the class II hydrophobins HFBI and HFBII from *Trichoderma reesei* have been solved [15], [16]. In addition, the structure of the class I EAS protein from *Neurospora crassa* has been determined by NMR [5]. These studies indicate that all hydrophobins share a similar small β-structured core that is dictated by the presence of the four disulfide bonds and that the proteins have large exposed hydrophobic surface regions that give rise to their high surface activity. The structures of the class I hydrophobins DewA (*Aspergillus nidulans*) and Mpg1 (*M. grisea*) and the class II hydrophobin from *N. crassa*, as well as the secondary structure of the class I hydrophobins RodA and RodB from *Aspergillus fumigatus* obtained through the analysis of their backbone NMR chemical shifts, are consistent with this (J. I. Guijarro and M. Sunde, unpublished data). Monolayer formation by class II hydrophobins does not appear to be associated with major conformational changes. In contrast, biophysical analysis of SC3 from *S. commune* and EAS indicate that rodlet formation is associated with significant structural rearrangements, in some cases involving helical intermediates, but always to a final rodlet form with high β-sheet content and amyloid characteristics [5], [17, Reference S3 in Text S1]. Digestion and hydrogen-deuterium exchange experiments with SC3 [18] indicated that the Cys3–Cys4 loop is important for adhesion to hydrophobic surfaces and may directly participate in the formation of rodlets. However, truncation [19] and systematic site-directed mutagenesis [20] experiments with EAS have shown that the Cys3–Cys4 loop is not involved in rodlet formation and that the Cys7–Cys8 loop region is crucial for auto-assembly, suggesting that the variability of the sequences of class I hydrophobins may translate into different mechanisms of rodlet formation [18]. Nevertheless, the surface tension seems to be the driving force to recruit class I hydrophobins to the air–water interface where the structural changes from the soluble form to the rodlet conformation take place [21].

## Hydrophobins and Fungus–Host Interactions

The surface rodlet-layer has a critical role in masking the immunogenicity of airborne fungal spores [22]. By covering the spore surface, the rodlet-layer imparts immunological inertness to the spores and ensures that pathogen-associated molecular patterns (PAMPs) are not recognized by innate and adaptive immune cells, thus preventing the activation of host immune system, inflammation, and tissue damage [22], [23], [24], [25, Reference S4 in Text S1]. Several lines of evidence suggest that the rodlet-layer, which covers the spores of both pathogenic and non-pathogenic fungal species, prevents immune recognition [22], [23], [25] (Figure 1). In opportunistic pathogen *A. fumigatus*, the rodlet-layer made up of RodA imparts resistance to NETosis (a process associated with disruption of neutrophil-membranes and release of a mixture of nuclear DNA with a granular content that acts as a neutrophil extracellular trap [NET]) and killing by alveolar macrophages [23], [26]. However, removal of *RODA* and *RODB* did not affect pathogenicity of *A. fumigatus* [Reference S5 in Text S1].

In plant-/entomo-pathogenic fungi, hydrophobins are also described as pathogenicity factors, but their precise role in fungal virulence remains to be understood. In the rice blast fungus *M. grisea*, the hydrophobin Mpg1 is suggested to function as a developmental sensor for appresorium formation, since it is involved in the interaction with hydrophobic leaf surfaces necessary for establishing the pathogenicity [13]. Deletion of the *MPG1* gene resulted in a mutant of *M. grisea* with reduced virulence; the deletion of another hydrophobin gene in *M. grisea*, *MHP1*, led also to a loss of viability and a reduced capacity to infect and colonize a susceptible rice cultivar [27]. In *Beauveria bassiana*, the non-specific hydrophobic interaction between the fungal spore coat hydrophobin and the insect epicuticle is involved in establishing the pathogenicity of the fungus [28].

## Prospective Applications of Hydrophobins

The potential applications of hydrophobins rely on their ability to reverse the hydrophilic-hydrophobic character of a surface and/or their surfactant capacity. Several biotechnological applications of hydrophobins have been proposed [29, Reference S6–S12 in Text S1]. However, the large-scale applications of hydrophobins might be difficult to implement due to the production cost of recombinant proteins and/or the large-scale requirements of the proteins. In contrast, in the pharmaceutical or in the nanotechnology industry, where the returns of investment are high, it is possible to envisage a potential development for these proteins. For example, the foam and air-/oil-filled emulsion-forming capacity of hydrophobins has been exploited in protecting nanoparticles and drug formulations [30, Reference S13–S16 in Text S1] (Figure 1). From a therapeutic point of view, the degradation-resistance and immunologically inert properties of hydrophobins could be used to generate hydrophobin-based nanoparticles with embedded therapeutic proteins and molecules that have to be slowly released within the host or transported to a specific body location without being recognized by the host immune system.

Many questions, however, remain unsolved in the study of hydrophobins: for instance, how is the 3D rodlet-structure organized? How are hydrophobins transported to the cell surface? How is the rodlet-layer attached to the spore surface? What are the signals that trigger germination of the spores covered by a rodlet layer? Addressing these questions will reveal the mechanism by which hydrophobins accomplish their multiple roles in the fungal life cycle.

## Supporting Information

Text S1 Supplementary references S1–S16.
(DOC)

## References

[1] Wessels J, De Vries O, Asgeirsdottir SA, Schuren F (1991). Hydrophobin Genes Involved in Formation of Aerial Hyphae and Fruit Bodies in Schizophyllum.. Plant Cell.

[2] Kodani S, Lodato MA, Durrant MC, Picart F, Willey JM (2005). SapT, a lanthionine-containing peptide involved in aerial hyphae formation in the streptomycetes.. Mol Microbiol.

[3] Sunde M, Kwan AH, Templeton MD, Beever RE, Mackay JP (2008). Structural analysis of hydrophobins.. Micron.

[4] Littlejohna KA, Hooleyb P, Cox PW (2012). Bioinformatics predicts diverse Aspergillus hydrophobins with novel properties.. Food Hydrocolloids.

[5] Kwan AH, Winefield RD, Sunde M, Matthews JM, Haverkamp RG (2006). Structural basis for rodlet assembly in fungal hydrophobins.. Proc Natl Acad Sci U S A.

[6] Wessels JG (1997). Hydrophobins: proteins that change the nature of the fungal surface.. Adv Microb Physiol.

[7] Jensen BG, Andersen MR, Pedersen MH, Frisvad JC, Sondergaard I (2010). Hydrophobins from Aspergillus species cannot be clearly divided into two classes.. BMC Res Notes.

[8] Kershaw MJ, Wakley G, Talbot NJ (1998). Complementation of the mpg1 mutant phenotype in *Magnaporthe grisea* reveals functional relationships between fungal hydrophobins.. EMBO J.

[9] Wang X, Shi F, Wosten HA, Hektor H, Poolman B (2005). The SC3 hydrophobin self-assembles into a membrane with distinct mass transfer properties.. Biophys J.

[10] Latgé JP, Cole GT, Horis berger M, Prévost MC (1986). Ultrastructure and chemical composition of the ballistospore wall of *Conidiobolus obscurus*.. Exp Mycol.

[11] De Groot PW, Schaap PJ, Sonnenberg AS, Visser J, Van Griensven LJ (1996). The Agaricus bisporus hypA gene encodes a hydrophobin and specifically accumulates in peel tissue of mushroom caps during fruit body development.. J Mol Biol.

[12] Kazmierczak P, Kim DH, Turina M, Van Alfen NK (2005). A hydrophobin of the chestnut blight fungus, *Cryphonectria parasitica*, is required for stromal pustule eruption.. Eukaryot Cell.

[13] Talbot NJ, Kershaw MJ, Wakley GE, De Vries O, Wessels J (1996). MPG1 Encodes a fungal hydrophobin involved in surface interactions during infection-related development of *Magnaporthe grisea*.. Plant Cell.

[14] Scherrer S, De Vries OM, Dudler R, Wessels JG, Honegger R (2000). Interfacial self-assembly of fungal hydrophobins of the lichen-forming ascomycetes *Xanthoria parietina* and *X. ectaneoides*.. Fungal Genet Biol.

[15] Hakanpaa J, Paananen A, Askolin S, Nakari-Setala T, Parkkinen T (2004). Atomic resolution structure of the HFBII hydrophobin, a self-assembling amphiphile.. J Biol Chem.

[16] Hakanpaa J, Szilvay GR, Kaljunen H, Maksimainen M, Linder M (2006). Two crystal structures of Trichoderma reesei hydrophobin HFBI–the structure of a protein amphiphile with and without detergent interaction.. Protein Sci.

[17] de Vocht ML, Reviakine I, Ulrich WP, Bergsma-Schutter W, Wosten HA (2002). Self-assembly of the hydrophobin SC3 proceeds via two structural intermediates.. Protein Sci.

[18] Wang X, Permentier HP, Rink R, Kruijtzer JA, Liskamp RM (2004). Probing the self-assembly and the accompanying structural changes of hydrophobin SC3 on a hydrophobic surface by mass spectrometry.. Biophys J.

[19] Kwan AH, Macindoe I, Vukasin PV, Morris VK, Kass I (2008). The Cys3–Cys4 loop of the hydrophobin EAS is not required for rodlet formation and surface activity.. J Mol Biol.

[20] Macindoe I, Kwan AH, Ren Q, Morris VK, Yang W (2012). Self-assembly of functional, amphipathic amyloid monolayers by the fungal hydrophobin EAS.. Proc Natl Acad Sci U S A.

[21] Morris VK, Ren Q, Macindoe I, Kwan AH, Byrne N (2011). Recruitment of class I hydrophobins to the air:water interface initiates a multi-step process of functional amyloid formation.. J Biol Chem.

[22] Aimanianda V, Bayry J, Bozza S, Kniemeyer O, Perruccio K (2009). Surface hydrophobin prevents immune recognition of airborne fungal spores.. Nature.

[23] Bruns S, Kniemeyer O, Hasenberg M, Aimanianda V, Nietzsche S (2010). Production of extracellular traps against *Aspergillus fumigatus* in vitro and in infected lung tissue is dependent on invading neutrophils and influenced by hydrophobin RodA.. PLoS Pathog.

[24] Hohl TM, Van Epps HL, Rivera A, Morgan LA, Chen PL (2005). *Aspergillus fumigatus* triggers inflammatory responses by stage-specific beta-glucan display.. PLoS Pathog.

[25] Dagenais TR, Giles SS, Aimanianda V, Latge JP, Hull CM (2010). Aspergillus fumigatus LaeA-mediated phagocytosis is associated with a decreased hydrophobin layer.. Infect Immun.

[26] Paris S, Debeaupuis JP, Crameri R, Carey M, Charles F (2003). Conidial hydrophobins of *Aspergillus fumigatus*.. Appl Environ Microbiol.

[27] Kim S, Ahn IP, Rho HS, Lee YH (2005). MHP1, a Magnaporthe grisea hydrophobin gene, is required for fungal development and plant colonization.. Mol Microbiol.

[28] Zhang S, Xia YX, Kim B, Keyhani NO (2011). Two hydrophobins are involved in fungal spore coat rodlet layer assembly and each play distinct roles in surface interactions, development and pathogenesis in the entomopathogenic fungus, *Beauveria bassiana*.. Mol Microbiol.

[29] Linder MB, Szilvay GR, Nakari-Setala T, Penttila ME (2005). Hydrophobins: the protein-amphiphiles of filamentous fungi.. FEMS Microbiol Rev.

[30] Valo HK, Laaksonen PH, Peltonen LJ, Linder MB, Hirvonen JT (2010). Multifunctional hydrophobin: toward functional coatings for drug nanoparticles.. ACS Nano.

