# Supplementary material for: Hydrophobins—Unique Fungal Proteins
Source: PLoS Pathog. 2012 May 31;8(5):e1002700. doi: 10.1371/journal.ppat.1002700 (PMC3364958; doi:10.1371/journal.ppat.1002700)
Supplement: Text S1 — Supplementary references S1–S16. (DOC) [file ppat.1002700.s001.doc]

**SUPPORTING INFORMATION**

**Text S1**

S1. Wosten HA, Asgeirsdottir SA, Krook JH, Drenth JH, Wessels JG (1994) The fungal hydrophobin Sc3p self-assembles at the surface of aerial hyphae as a protein membrane constituting the hydrophobic rodlet layer. Eur J Cell Biol 63: 122-129.

S2. Kim S, Ahn IP, Rho HS, Lee YH (2005) MHP1, a Magnaporthe grisea hydrophobin gene, is required for fungal development and plant colonization. Mol Microbiol 57: 1224-1237.

S3. de Vocht ML, Scholtmeijer K, van der Vegte EW, de Vries OM, Sonveaux N, et al. (1998) Structural characterization of the hydrophobin SC3, as a monomer and after self-assembly at hydrophobic/hydrophilic interfaces. Biophys J 74: 2059-2068.

S4. Steele C, Rapaka RR, Metz A, Pop SM, Williams DL, et al. (2005) The beta-glucan receptor dectin-1 recognizes specific morphologies of Aspergillus fumigatus. PLoS Pathog 1: e42.

S5. Thau N, Monod M, Crestani B, Rolland C, Tronchin G, et al. (1994) rodletless mutants of Aspergillus fumigatus. Infect Immun 62: 4380-4388.

S6. Zhao ZX, Wang HC, Qin X, Wang XS, Qiao MQ, et al. (2009) Self-assembled film of hydrophobins on gold surfaces and its application to electrochemical biosensing. Colloids Surf B Biointerfaces 71: 102-106.

S7. Wang X, Wang H, Huang Y, Zhao Z, Qin X, et al. (2010) Noncovalently functionalized multi-wall carbon nanotubes in aqueous solution using the hydrophobin HFBI and their electroanalytical application. Biosens Bioelectron 26: 1104-1108.

S8. Kottmeier K, Ostermann K, Bley T, Rodel G (2011) Hydrophobin signal sequence mediates efficient secretion of recombinant proteins in Pichia pastoris. Appl Microbiol Biotechnol 91: 133-141.

S9. Mustalahti E, Saloheimo M, Joensuu JJ (2011) Intracellular protein production in Trichoderma reesei (Hypocrea jecorina) with hydrophobin fusion technology. N Biotechnol. doi:10.1016/j.nbt.2011.09.006 (*Epub ahead of print*)

S10. Niu B, Wang D, Yang Y, Xu H, Qiao M (2011) Heterologous expression and characterization of the hydrophobin HFBI in Pichia pastoris and evaluation of its contribution to the food industry. Amino Acids. doi: 10.1007/s00726-011-1126-5 (*Epub ahead of print*)

S11. Deckers SM, Lorgouilloux Y, Gebruers K, Baggerman GA, Michiels C, et al. (2011) Dynamic Light Scattering (DLS) as a Tool to Detect CO2-Hydrophobin Structures and Study the Primary Gushing Potential of Beer. J Am Soc Brew Chem 69: 144-149.

S12. Shokribousjein Z, Deckers SM, Gebruers K, Lorgouilloux Y, Baggerman G, et al. (2011) Hydrophobins, beer foaming and gushing Cerevisia 35: 85-101.

S13. Basheva ES, Kralchevsky PA, Christov NC, Danov KD, Stoyanov SD, et al. (2011) Unique Properties of Bubbles and Foam Films Stabilized by HFBII Hydrophobin. Langmuir 27:2382-2392.

S14.Valo H, Kovalainen M, Laaksonen P, Hakkinen M, Auriola S, et al. (2011) Immobilization of protein-coated drug nanoparticles in nanofibrillar cellulose matrices-Enhanced stability and release. J Control Release 156:390-397.

S15. Haas Jimoh Akanbi M, Post E, Meter-Arkema A, Rink R, Robillard GT, et al. (2010) Use of hydrophobins in formulation of water insoluble drugs for oral administration. Colloids Surf B Biointerfaces 75: 526-531.

S16. Sarparanta MP, Bimbo LM, Makila EM, Salonen JJ, Laaksonen PH, et al. (2012) The mucoadhesive and gastroretentive properties of hydrophobin-coated porous silicon nanoparticle oral drug delivery systems. Biomaterials 33:3353-3362.
